# Draft Genome Sequence of the Putative Marine Pathogen *Thalassobius* sp. I31.1

**DOI:** 10.1128/MRA.01431-18

**Published:** 2019-02-07

**Authors:** Hilary J. Ranson, Jason LaPorte, Edward Spinard, Andrei Y. Chistoserdov, Marta Gomez-Chiarri, David R. Nelson, David C. Rowley

**Affiliations:** a Department of Biomedical and Pharmaceutical Sciences, University of Rhode Island, Kingston, Rhode Island, USA; b Department of Cell and Molecular Biology, University of Rhode Island, Kingston, Rhode Island, USA; c Department of Biology, University of Louisiana at Lafayette, Lafayette, Louisiana, USA; d Department of Fisheries, Animal and Veterinary Sciences, University of Rhode Island, Kingston, Rhode Island, USA; University of Maryland School of Medicine

## Abstract

*Thalassobius* sp. I31.1 is a putative pathogen involved in epizootic shell disease in the American lobster (Homarus americanus). We report here the draft genome sequence for *Thalassobius* sp. I31.1 and provide insight into its metabolism and links to environmental pollutant degradation.

## ANNOUNCEMENT

*Thalassobius* sp. I31.1 is a Gram-negative, aerobic, motile, rod-shaped bacterium. This genus is of the class *Alphaproteobacteria*, family *Rhodobacteraceae*, and order *Rhodobacterales*. *Thalassobius* sp. I31.1 was isolated from a lesion on the carapace of an American lobster (Homarus americanus) (1). Lesions are the characteristic symptom of epizootic shell disease (ESD), and *Thalassobius* has been indicated as a putative pathogen in lesion formation (1). Although *Thalassobius* strains may be unable to utilize various carbohydrates for growth (2), they can possess the ability to degrade the potential carcinogen phthalate, a chemical widely used in the manufacture of plastics, lubricants, and textiles (3). Such strains contain genes required for poly-β-hydroxybutyrate (PHB) synthesis and genes relating to the degradation of phenylacetates, an abundant class of environmental pollutants (4).

*Thalassobius* sp. I31.1 was grown in artificial seawater (Instant Ocean) supplemented with yeast extract (1 g/liter) and peptone (5 g/liter) at 25°C on an elliptical shaker (New Brunswick) for 48 h. Genomic DNA was isolated with the Promega Wizard DNA purification kit, and DNA was resuspended in 2 mM Tris-HCl buffer (Bio Basic). DNA was quantified with a NanoDrop 1000 spectrophotometer (ND-1000) and checked for quality on a 1% agarose gel stained with ethidium bromide. DNA was sequenced on an Illumina MiSeq sequencer at the Genomics and Sequencing Center at the University of Rhode Island. Total genomic DNA was sheared with sonication (Covaris S220), and libraries were prepared with the SMARTer PrepX DNA Library kit on a SMARTer Apollo system (TaKaRa Bio, USA). Reads were trimmed with the CLC Genomic Workbench (version 9.5.3) for quality, ambiguous base pairs, adaptors, duplicates, and size with default parameters, resulting in 2,277,834 paired-end reads. The draft genome was assembled using the *de novo* assembly algorithm of SPAdes assembler (version 3.1.1) (5). Contigs with a coverage of >44 reads were processed with the CLC Microbial Genome Finishing module. The completed draft genome is composed of 65 contigs (*N*_50_ contig length, 182,141 bp), averaging 57,385 bp in size (total genome, 3,729,993 bp), with an average G+C content of 55.1%. The draft genome was annotated with the Rapid Annotations using Subsystem Technology (RAST) server and resulted in 3,749 open reading frames (6).

The annotated draft genome of *Thalassobius* sp. I31.1 encodes type I and II/IV secretion systems. RAST annotation identified a suite of *paa* genes involved in phenylacetate degradation, two alkane monooxygenase genes, *phbF*, a transcriptional regulator involved in PHB synthesis (7), and one toluenesulfonate dehydrogenase. The RAST-annotated draft genome was entered into Antibiotics and Secondary Metabolite Analysis Shell (antiSMASH) for secondary metabolite biosynthesis gene cluster analysis (8). Six clusters for secondary metabolism were identified, including those for homoserine lactone, bacteriocin, and lantipeptide-bacteriocin biosynthesis.

### Data availability.

This whole-genome shotgun project has been deposited at DDBJ/EMBL/GenBank under the accession number PWAA00000000. The version described in this paper is the first version, PWAA01000000. The SRA/DRA/ERA accession number is PRJNA437474.
